# Lower socioeconomic status associated with higher tuberculosis rate in South Korea

**DOI:** 10.1186/s12890-023-02713-z

**Published:** 2023-10-31

**Authors:** Seong-Woo Choi, Jeong-Ja Im, Sang-Eun Yoon, Seo-Hee Kim, Jun-Hwi Cho, So-Jung Jeong, Kyung-Ae Park, Young-Sung Moon

**Affiliations:** 1 Department of Preventive Medicine, Chosun University Medical School, 309, Pilmun-daero, Dong-gu, Gwangju, 61452 Republic of Korea; 2 Department of Public Health, Graduate School of Chosun University, 309, Pilmun-daero, Dong-gu, Gwangju, 61452 Republic of Korea

**Keywords:** Income, Education, Socioeconomic status, Tuberculosis

## Abstract

**Background:**

Tuberculosis is an infectious disease influenced by social factors rather than a simple infectious disease. In this study, we investigated the relationship between tuberculosis rates and socioeconomic status.

**Methods:**

This study was conducted using data of the 49,483 participants of the Korean National Health and Nutrition Examination Survey (KNHANES) VI–VIII (2013–2021). The relationships between tuberculosis rates and the quartiles of monthly household income and education level were examined using a multivariate logistic regression analysis.

**Results:**

The KNHANES data revealed that the prevalence of tuberculosis as substantially related to monthly household income (odds ratio [OR], 6.0; 95% confidence interval [CI], 1.1–32.0 for lowest vs. highest incomes) and education level (OR, 3.8; 95% CI, 1.2–12.0 for 10–12 years vs. ≥13 years; OR, 4.1; 95% CI, 1.2–14.8 for ≤ 6 years vs. ≥13 years). Furthermore, current tuberculosis treatment was significantly related to monthly household income and education level.

**Conclusion:**

There were substantial correlations between tuberculosis rates and socioeconomic status in South Korea.

## Introduction

**Fig. 1:**
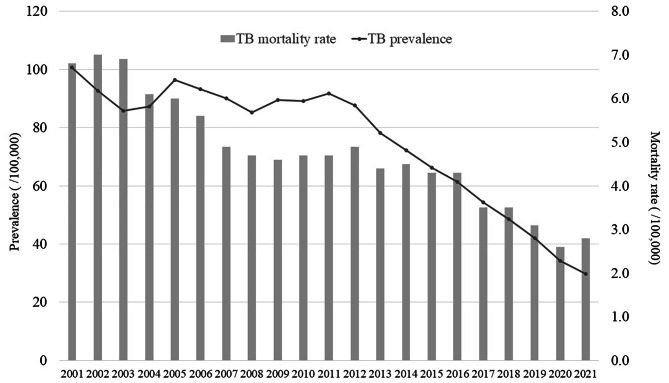
Age-standardized prevalence and mortality rate of tuberculosis in South Korea, 2001–2021

Tuberculosis (TB) is an infectious disease that is one of the leading causes of death throughout the world. Prior to the COVID-19 pandemic, TB had the highest mortality rate for a single infectious disease. According to the World Health Organization (WHO), there were  9.87 million tuberculosis cases and  1.49 million related deaths worldwide in 2020 [1]. In particular, multidrug-resistant TB has significantly increased the burden of the disease. Korea has one of the highest TB disease burdens among the Organization for Economic Co-operation and Development (OECD) countries. The TB mortality rate fell from 6.8 to 100,000 in 2001 to 2.8 per 100,000 in 2021, while the prevalence rate fell from 100.6 to 100,000 in 2001 to 29.7 per 100,000 in 2021 [2] (Fig. 1); however, these rates remain the highest among OECD countries.

International organizations have attempted to break the transmission chains by identifying cases early and providing effective treatment through a TB control program [3]. However, these TB-treatment strategies have not proven to be sufficiently effective and, on a global scale, TB elimination remains a major challenge. This is because TB is not a simple infectious disease but an infectious disease influenced by social factors [3]. In other words, social determinants of health influence all aspects of TB, including exposure, diagnosis, treatment, and recovery [4]. Previous ecological studies have found links between high TB rates in different areas and low levels of education, high levels of poverty, and income inequality [5–7]. In individual-level studies, authors determined economic deprivation, homelessness, prison terms, injection of drugs, HIV/AIDS, and migration from high TB-rate countries as risk factors for TB [8, 9].

Korea has numerous distinct TB-related factors. Despite being a high-income country, it has a high incidence of TB and a few HIV/AIDS patients. Surprisingly, few studies have been conducted on the connection between socioeconomic status (SES) and TB in Korea. Therefore, we examined the relationship between TB and SES using the representative health data of Korea extracted from the Korea National Health and Nutrition Examination Survey (KNHANES) 2013–2021.

## Methods

### Subjects

This study was conducted using data extracted from KNHANES VI–VIII (2013–2021). KNHANES data have previously been published [10]. The Korea Centers for Disease Control and Prevention (KCDCP) conducted KNHANES on an annual basis (VI, 2013–2015; VII, 2016–2018; and VIII, 2019–2021), employing a nationally representative cross-sectional design. A total of 69,776 subjects were enrolled in KNHANES VI–VIII (2013–2021) (VI [2013: 8,018; 2014: 7,550; 2015: 7,380], VII [2016: 8,150; 2017: 8,127; 2018: 7,992], and VIII [2019: 8,110; 2020: 7,359; 2021: 7,090]). A total of 20,293 people were excluded; 13,662 were under the age of 19, 6,207 did not have tuberculosis data, and 424 did not have sociodemographic or behavioral data. Finally, 49,483 participants were examined in this study. All participants gave their written permission for their data to be used. Our research was carried out following the guidelines of the Declaration of Helsinki. The KCDCP ethics committee authorized the study protocol (2013-07CON-03-4 C, 2014-12EXP-03-5 C, 2015-01-02-6 C, 2018-01-03-P-A, 2018-01-03-1 C-A, 2018-01-03-2 C-A).

### Study measurements

Individual interviews were conducted by trained researchers using a structured questionnaire. A person who answered “yes” to the question “being prevalent with TB” was defined as a patient, and a person who answered “yes” to the question “being treated with TB” was defined as a currently-treated TB patient. The monthly household income was divided into four quartiles (highest, medium-highest, medium-lowest, and lowest). The education level was classified as ≤ 6, 7–9, 10–12, and ≥ 13 years. The participants’ weight (to the nearest 0.1 kg) and height (to the nearest 0.1 cm) were measured while wearing light clothing and socks. Body mass index (BMI) was calculated by dividing the weight in kilograms by the height in meter squared. The marital status was classified as unmarried or married, and the residential area was classified as urban or rural. Current smokers were those who smoked regularly or occasionally, and current drinkers were those who had one or more drinking experiences in the previous month. Walking for more than thirty minutes at a time and more than five times per week was characterized as a physical activity. A health checkup within the last two years was defined as a case in which a health checkup was performed within the last two years; a cancer examination within the last two years was defined as a case in which a cancer examination was performed within the last two years.

### Statistical analysis

The survey responses were weighted using a multilevel, multiple, probability-sampling design to represent nationally representative prevalence estimates of the Korean population. The estimates were derived by considering the primary sampling unit, stratification variables, and sampling weights. The data were expressed as an estimated percentage, with a 95% confidence interval [CI]. The distributions of each variable according to the quartiles of monthly household income and education level were examined using cross tabulation. The relationships between TB and the quartiles of monthly household income and education level were examined using a multivariate logistic regression analysis. The crude odds ratio (OR) and adjusted OR for gender, age, BMI, survey year, marital status, residence area, current smoking, drinking, physical activity, health checkup, and cancer examination over the previous two years are presented herein. P-values < 0.05 were deemed statistically significant. Statistical analysis was conducted using SPSS ver. 22.0.

## Results

### Baseline characteristics

The TB prevalence rate (per 100,000) was 64.8 and the current TB-treatment rate (per 100,000) is 55.3. Monthly household income was in the range of 15.0% for the lowest, 23.8% for the medium-lowest, 29.3% for the medium-highest, and 31.9% for the highest. The education level was 14.4% for ≤ 6 years, 8.5 for 7–9 years, 36.8% for 10–12 years, and 40.3% for ≥ 13 years. 20.9% of all subjects currently smoked and 57.6% had consumed alcohol in the previous month; 65.4% had a health checkup in the previous 2 years and 52.8% had a cancer examination in the previous 2 years (Table 1).

**Table 1:** Baseline characteristics of subjects

Variables	e%(95% CI)
TB prevalence (/100,000)	64.8 (44.9–93.4)
Current TB treatment (/100,000)	55.3 (36.7–83.2)
Gender	
Male	49.4 (49.0 − 49.8)
Female	50.6 (50.2 − 51.0)
Age (years)	
≤ 40	38.2 (37.4 − 38.9)
41–64	45.5 (44.8 − 46.1)
≥ 65	16.4 (15.8 − 16.9)
Body mass index (kg/m^2^)	
< 18.5	4.4 (4.1 − 4.6)
18.5–22.9	38.3 (37.7 − 38.8)
23.0–24.9	22.6 (22.2 − 23.1)
≥ 25.0	34.7 (34.2 − 35.3)
Survey year	
KNHANES VI (2013–2015)	31.1 (30.4 − 31.9)
KNHANES VII (2016–2018)	34.1 (33.3 − 35.0)
KNHANES VIII (2019–2021)	34.8 (33.9 − 35.7)
Monthly household income	
Lowest	15.0 (14.4 − 15.6)
Medium-lowest	23.8 (23.1 − 24.5)
Medium-highest	29.3 (28.6 − 30.1)
Highest	31.9 (30.8 − 32.9)
Education (year)	
≤ 6	14.4 (13.9 − 15.0)
7–9	8.5 (8.2 − 8.9)
10–12	36.8 (36.1 − 37.4)
≥ 13	40.3 (39.4 − 41.2)
Marital status	
Single	34.0 (33.3 − 34.7)
Married	66.0 (65.3 − 66.7)
Residential area	
Urban	84.3 (82.6 − 85.8)
Rural	15.7 (14.2 − 17.4)
Number of household numbers	
1	10.1 (9.6–10.7)
2–4	80.1 (79.5–80.8)
≥ 5	9.8 (9.2–10.3)
Current smoking	20.9 (20.4 − 21.4)
Alcohol intake	57.6 (57.0 − 58.2)
^a^Physically active	45.9 (45.3 − 46.6)
^b^Health examination during past 2 years	65.4 (64.8 − 65.9)
^c^Cancer examination during past 2 years	52.8 (52.1 − 53.4)

All values are given as number and estimated percentage (95% confjdence interval).

CI; confidence interval, TB; tuberculosis, KNHANES; the Korea national health and nutrition examination survey

^a^Physically active was indicated as ‘yes’ when the subject walked for more than 30 min at a time and more than five times per week.

^b^Health examination during past 2 years was indicated as ‘yes’ when the subject had undergone health checkup within the prior 2 years.

^c^Cancer examination during past 2 years was indicated as ‘yes’ when the subject had undergone screening within the prior 2 years for any kind of cancer such as lung cancer, stomach cancer, and breast cancer etc.

### Characteristics of subjects by the quartile of monthly household income

The survey year, age, BMI, and current smoking differed significantly based on monthly household income quartiles. Furthermore, being a male, educated, urban dweller, alcohol intake, physical activity, health checkup, and cancer examination during the previous 2 years significantly increased with increasing monthly household income, whereas TB prevalence, current TB treatment, and being unmarried significantly decreased (Table 2).

**Table 2:** Characteristics of subjects according to the quartile of monthly household income

Variables	Monthly household income				
	Lowest	Medium-lowest	Medium-highest	Highest	*P*
TB prevalence (/100,000)	181.7(105.3-313.6)	63.0(30.7-129.6)	61.1(27.8-134.2)	14.3(4.1–49.5)	< 0.001
Current TB treatment (/100,000)	140.2(73.5-267.3)	69.6(35.3–137.0)	52.8(22.0-126.9)	6.9(1.0-48.8)	< 0.001
Gender					< 0.001
Male	42.8(41.6–43.9)	48.1(47.2–48.9)	50.9(50.1–51.7)	52.1(51.4–52.9)	
Female	57.2(56.1–58.4)	51.9(51.1–52.8)	49.1(48.3–49.9)	47.9(47.1–48.6)	
Age (years)					< 0.001
≤ 40	20.2(18.6–21.8)	37.4(36.1–38.7)	43.8(42.6–44.9)	42.2(41.1–43.3)	
41–64	30.2(29.0-31.5)	43.7(42.6–44.8)	47.2(46.1–48.3)	52.3(51.3–53.4)	
≥ 65	49.7(48.0-51.3)	18.9(18.0-19.8)	9.0(8.5–9.6)	5.5(5.1-6.0)	
Body mass index (kg/m^2^)					< 0.001
< 18.5	4.6(4.0-5.2)	4.7(4.2–5.2)	4.0(3.6–4.4)	4.4(4.0-4.8)	
18.5–22.9	36.2(34.9–37.5)	36.7(35.7–37.8)	38.6(37.7–39.6)	40.0(39.1–41.0)	
23.0–24.9	22.6(21.6–23.6)	22.7(21.8–23.6)	22.0(21.2–22.8)	23.1(22.4–23.9)	
≥ 25.0	36.7(35.4–38.0)	35.9(34.8–37.0)	35.4(34.4–36.4)	32.5(31.5–33.4)	
Survey year					0.039
KNHANES VI (2013–2015)	31.7(29.9–33.5)	32.4(30.9–33.9)	31.3(29.8–32.7)	29.7(28.0-31.5)	
KNHANES VII (2016–2018)	35.8(33.9–37.7)	33.8(32.3–35.3)	34.3(32.8–35.8)	33.5(31.6–35.3)	
KNHANES VIII (2019–2021)	32.5(30.7–34.3)	33.9(32.4–35.4)	34.4(32.9–36.0)	36.8(34.8–38.8)	
Education (year)					< 0.001
≤ 6	45.3(43.7–47.0)	17.2(16.4–18.1)	8.0(7.5–8.5)	3.6(3.3-4.0)	
7–9	12.8(12.0-13.6)	11.7(11.0-12.4)	7.8(7.3–8.4)	4.7(4.4–5.2)	
10–12	28.8(27.3–30.3)	40.6(39.4–41.7)	40.4(39.3–41.5)	34.3(33.2–35.4)	
≥ 13	13.1(12.1–14.1)	30.5(29.3–31.7)	43.8(42.6–45.1)	57.3(56.1–58.5)	
Marital status					< 0.001
Single	51.5(49.9–53.0)	32.9(31.7–34.1)	30.3(29.2–31.4)	30.1(29.0-31.1)	
Married	48.5(47.0-50.1)	67.1(65.9–68.3)	69.7(68.6–70.8)	69.9(68.9–71.0)	
Residential area					< 0.001
Urban	74.7(71.9–77.3)	82.1(80.0–84.0)	85.9(83.9–87.6)	88.9(87.1–90.4)	
Rural	25.3(22.7–28.1)	17.9(16.0–20.0)	14.1(12.4–16.1)	11.1(9.6–12.9)	
Number of household numbers					< 0.001
1	29.2(27.7-3)0.7	9.6(8.9–10)0.4	6.4(5.7-7.)1	5.0(4.4-5.)5	
2–4	65.2(63.6-6)6.7	79.8(78.6–81.0)	82.6(81.5–83.7)	85.1(84.1–86.1)	
≥ 5	5.7(4.8–6.7)	10.6(9.6-1)1.6	11.0(10.0–12.0)	9.9(9.0–10)0.9	
Smoking	19.0(17.9–20.2)	21.6(20.7–22.6)	22.9(22.0-23.9)	19.5(18.6–20.3)	< 0.001
Alcohol intake	41.1(39.7–42.5)	54.8(53.7–55.9)	60.6(59.5–61.7)	64.7(63.7–65.6)	< 0.001
Physically active^a^	41.9(40.5–43.3)	45.5(44.4–46.6)	45.9(44.8–47.0)	48.3(47.2–49.4)	< 0.001
Health examination during past 2 years^b^	57.0(55.7–58.3)	60.2(59.0-61.3)	66.1(65.1–67.1)	72.5(71.5–73.4)	< 0.001
Cancer examination during past 2 years^c^	49.0(47.6–50.4)	50.1(48.9–51.3)	51.8(50.7–52.9)	57.5(56.5–58.6)	< 0.001

All values are given as number and estimated percentage (95% confjdence interval).

TB; tuberculosis, KNHANES; the Korea national health and nutrition examination survey

^a^Physically active was indicated as ‘yes’ when the subject walked for more than 30 min at a time and more than five times per week.

^b^Health examination during past 2 years was indicated as ‘yes’ when the subject had undergone health checkup within the prior 2 years.

^c^Cancer examination during past 2 years was indicated as ‘yes’ when the subject had undergone screening within the prior 2 years for any kind of cancer such as lung cancer, stomach cancer, and breast cancer etc.

### Characteristics of subjects by educational level

TB prevalence, current TB treatment, BMI, survey years, marital status, current smoking, physical activity, health checkup, and cancer examination over the previous 2 years varied significantly based on education levels. Furthermore, being a male, having a monthly household income, urban dweller, and drinking alcohol increased significantly with increasing education level (Table 3).

**Table 3:** Characteristics of subjects according to the quartile of education level

Variables	Education (year)				
	≤ 6	7–9	10–12	≥ 13	*P*
TB prevalence (/100,000)	176.2(107.0-290.2)	68.7(22.7-207.8)	74.4(37.9-145.6)	15.4(6.2–38.2)	< 0.001
Current TB treatment (/100,000)	147.6(84.2-258.6)	47.2(11.8-188.2)	68.0(33.3-138.6)	12.5(4.3–36.5)	< 0.001
Gender					< 0.001
Male	31.8(30.8–32.8)	47.1(45.6–48.7)	52.4(51.5–53.2)	53.4(52.7–54.2)	
Female	68.2(67.2–69.2)	52.9(51.3–54.4)	47.6(46.8–48.5)	46.6(45.8–47.3)	
Age (years)					< 0.001
≤ 40	1.3(1.0–1.)7	7.7(6.7-8.)8	42.7(41.7–43.7)	53.7(52.5–54.9)	
41–64	33.7(32.5–34.9)	62.9(61.2–64.5)	49.4(48.5–50.4)	42.3(41.2–43.5)	
≥ 65	65.0(63.8–66.2)	29.5(28.0–31.0)	7.9(7.4-8.)3	4.0(3.7-4.)3	
Body mass index (kg/m^2^)					< 0.001
< 18.5	2.7(2.4-3.)1	2.5(2.0–3.)1	4.7(4.4-5.)1	5.0(4.6-5.)4	
18.5–22.9	31.2(30.1–32.3)	33.6(32.1–35.2)	39.2(38.4–40.1)	40.9(40.1–41.7)	
23.0–24.9	25.4(24.5–26.5)	25.6(24.1–27.1)	22.4(21.6–23.1)	21.2(20.5–21.9)	
≥ 25.0	40.6(39.5–41.8)	38.3(36.7–39.9)	33.7(32.8–34.6)	32.9(32.1–33.7)	
Survey year					0.039
KNHANES VI (2013–2015)	36.5(34.8–38.2)	33.0(31.2–34.9)	32.3(31.1–33.5)	27.7(26.5–29.0)	
KNHANES VII (2016–2018)	34.6(32.9–36.3)	35.1(33.2–37.1)	32.7(31.5–33.9)	35.1(33.6–36.6)	
KNHANES VIII (2019–2021)	28.9(27.3–30.6)	31.9(30.0-33.8)	35.0(33.7–36.3)	37.2(35.7–38.8)	
Monthly household income					< 0.001
Lowest	47.3(45.9–48.7)	22.5(21.2–24.0)	11.8(11.0-12.6)	4.9(4.5-5.)3	
Medium-lowest	28.5(27.3–29.7)	32.8(31.2–34.4)	26.3(25.3–27.3)	18.0(17.1–18.9)	
Medium-highest	16.2(15.2–17.3)	27.0(25.4–28.6)	32.2(31.2–33.3)	31.9(30.8–32.9)	
Highest	8.0(7.3-8.)8	17.7(16.4–19.1)	29.7(28.5–31.0)	45.3(43.9–46.6)	
Marital status					< 0.001
Single	36.5(35.2–37.7)	22.0(20.7–23.5)	38.9(37.8–40.0)	31.2(30.1–32.4)	
Married	63.5(62.3–64.8)	78.0(76.5–79.3)	61.1(60.0-62.2)	68.8(67.6–69.9)	
Residential area					< 0.001
Urban	70.5(67.5–73.3)	78.3(75.7–80.7)	85.3(83.4–87.0)	89.5(87.9–90.9)	
Rural	29.5(26.7–32.5)	21.7(19.3–24.3)	14.7(13.0-16.6)	10.5(9.1-1)2.1	
Number of household numbers					< 0.001
1	20.9(19.8-2)1.9	11.6(10.7–12.7)	8.0(7.3-8.)8	7.9(7.2-8.)7	
2–4	72.3(71.1-7)3.4	79.8(78.4–81.2)	81.0(80.1–82.0)	82.2(81.2–83.1)	
≥ 5	6.8(6.1–7.6)	8.5(7.5-9.)7	10.9(10.2–11.8)	10.0(9.3-1)0.7	
Smoking	12.7(11.9–13.6)	21.4(20.0-22.8)	25.6(24.7–26.5)	19.5(18.7–20.2)	< 0.001
Alcohol intake	35.6(34.4–36.8)	50.8(49.1–52.4)	61.3(60.3–62.2)	63.5(62.7–64.4)	< 0.001
Physically active^a^	37.3(36.0-38.5)	42.7(41.1–44.3)	48.4(47.4–49.3)	47.5(46.6–48.5)	< 0.001
Health examination during past 2 years^b^	66.2(65.1–67.3)	69.1(67.5–70.6)	60.5(59.5–61.4)	68.8(67.9–69.6)	< 0.001
Cancer examination during past 2 years^c^	61.5(60.3–62.6)	63.9(62.2–65.6)	46.9(46.0-47.8)	52.7(51.6–53.8)	< 0.001

All values are given as number and estimated percentage (95% confjdence interval).

CI; confidence interval, TB; tuberculosis, KNHANES; the Korea national health and nutrition examination survey

^a^Physically active was indicated as ‘yes’ when the subject walked for more than 30 min at a time and more than five times per week.

^b^Health examination during past 2 years was indicated as ‘yes’ when the subject had undergone health checkup within the prior 2 years.

^c^Cancer examination during past 2 years was indicated as ‘yes’ when the subject had undergone screening within the prior 2 years for any kind of cancer such as lung cancer, stomach cancer, and breast cancer etc.

### The ORs for tuberculosis prevalence and treatment based on quartiles of monthly household income and educational level

Based on the adjustment for covariates, such as gender, age, BMI, survey year, marital status, residential area, number of household members, current smoking, alcohol intake, physical activity, health checkup, and cancer examination over the previous 2 years, TB prevalence was significantly correlated with monthly household income (OR, 6.0; 95% CI, 1.1–32.0 for lowest vs. highest) and education level (OR, 3.8; 95% CI, 1.2–12.0 for 10–12 years vs. ≥13 years; OR, 4.1; 95% CI, 1.2–14.8 for ≤ 6 years vs. ≥13 years). Moreover, current TB treatment was significantly correlated with monthly household income (OR, 8.1; 95% CI, 1.0–68.2 for medium-lowest vs. highest; OR, 12.3; 95% CI, 1.2–128.9 for lowest vs. highest) and education level (OR, 4.4; 95% CI, 1.2–16.4 for 10–12 years vs. ≥13 years; OR, 5.7; 95% CI, 1.3–24.4 for ≤ 6 years vs. ≥13 years) (Table 4).

**Table 4:** The ORs for tuberculosis by monthly household income and education level

		Crude	Adjusted^*^
		OR(95%CI)	OR (95%CI)
TB prevalence	Monthly household income		
	medium- highest/highest	4.3(1.0-18.7)	3.6(0.8–16.3)
	medium-lowest/highest	4.4(1.0-18.6)	3.1(0.7–14.1)
	lowest/highest	12.8(3.3–49.6)	6.0(1.1–32.0)
	Education (year)		
	10–12/≥13	4.8(1.5–15.1)	3.8(1.2–12.0)
	7–9/≥13	4.5(1.1–18.8)	2.6(0.5–12.8)
	≤ 6/≥13	11.5(4.0-32.6)	4.1(1.2–14.8)
Current TB treatment	Monthly household income		
	medium- highest/highest	7.7(0.9–66.1)	6.6(0.7–60.0)
	medium-lowest/highest	10.1(1.3–80.9)	8.1(1.0-68.2)
	lowest/highest	20.5(2.6-161.6)	12.3(1.2-128.9)
	Education (year)		
	10–12/≥13	5.5(1.5–19.9)	4.4(1.2–16.4)
	7–9/≥13	3.8(0.7–21.9)	2.8(0.4–20.5)
	≤ 6/≥13	11.9(3.5–40.2)	5.7(1.3–24.4)

^*^Adjusted by gender, age, BMI, survey year, marital status, residential area, number of household members, current smoking, alcohol intake, physically active, health checkup during the previous 2 years and cancer examination during the previous 2 years

## Discussion

This study assessed the relationship between SES and TB using KNHANES, a representative health dataset for Korea. Based on this study, we observed that the lower the monthly household income and education level, the greater the TB prevalence and the greater the TB treatment rate.

Our findings revealed a link between lower education levels and higher TB rates. In a study using data from the United States National TB Surveillance System from 1996 to 2005 [11], TB rates were highest among those with the least education. In a case-control study in England using the 2003–2012 UK Enhanced TB surveillance data, researchers found a link between higher education and lower TB rates [9], similar to our results. Education is known to be a strong predictor of future employment and earnings [12] as well as of increased awareness and/or increased ability to act on existing knowledge regarding healthy behavior [13, 14].

Our findings revealed a link between lower household income and higher TB rates. Previous research in low-income countries had yielded contradictory results. According to a case-control study conducted in Malawi, higher asset indexes is associated with lower TB rates [15]. However, other studies have not found a significant relationship between income and TB rates (5-glynn). High-income studies identified vulnerable groups of TB as economically disadvantaged people, the homeless, prisoners, and HIV/AIDS patients [3, 9]. The current global TB strategy focuses primarily on medical technologies, such as early case detection and effective treatment for the high-risk populations [16]. However, according to our findings, TB is a problem that is not limited to the vulnerable and marginalized groups. TB-related risks increase at a relatively constant rate with decreasing SES across the entire SES spectrum, similar to coronary heart disease, hypertension, cancer, and many chronic diseases [17]. This suggests that tuberculosis is not only a problem for high-risk groups but also a widespread societal health issue. In a 2006 Indian demographic health survey, TB prevalence increased linearly with the wealth quintile [18], similar to our findings.

There are several plausible explanations for the link between socioeconomic factors and TB. First, socioeconomic factors may expose people to TB, such as crowded and poorly ventilated work environments and housing. Second, malnutrition is a source of increased vulnerability. Third, increased risk factors include smoking, alcoholism, and comorbidities. Fourth, lack of access to health care results in delayed diagnosis and treatment.

Korea is a high-income country with a high TB incidence. The Korean government has implemented various tuberculosis eradication policies to reduce the prevalence of tuberculosis. The 2030 TB eradication plan was established in 2006, and the public private mix was expanded and implemented nationally in 2011. Furthermore, the government subsidized out-of-pocket TB-treatment costs in 2011 and the health coverage of latent TB-infected people was expanded in 2020, nearly eliminating the burden of out-of-pocket costs for tuberculosis testing and treatment [19]. Nonetheless, in this study, we observed differences in TB treatment rates based on SES. This is because, despite the free TB-treatment service provided by the public sector, patients bear direct costs associated with health seeking, indirect costs associated with lost income, and dissaving during illness, diagnosis, and treatment [20, 21]. These early costs are widespread, frequently severe, and have been linked to negative TB-treatment outcomes such as treatment failure, no follow-ups, or death.

Our research was based on extensive national health data. Despite the extensive register data of this study, it has some limitations. First, its cross-sectional design precludes drawing conclusions about the direction of the observed association between the TB rates and SES. Second, confounding effects may remain due to uncontrolled factors such as diabetes or cancer. Third, while KNHANES is a household-visit survey, it can under report cases of TB because it excludes high-risk populations, such as the homeless, inmates, and foreign workers. In conclusion, there were significant associations between TB rates and SES in South Korea.

## Data Availability

The datasets generated and/or analysed during the current study are available in the KNHANES web site, https://knhanes.kdca.go.kr/knhanes/sub03/sub03_02_05.do.
